# Lasting Effects of Low to Non-Lethal Radiation Exposure during Late Gestation on Offspring’s Cardiac Metabolism and Oxidative Stress

**DOI:** 10.3390/antiox10050816

**Published:** 2021-05-20

**Authors:** Ashley S. Nemec-Bakk, Sarah Niccoli, Caitlund Davidson, Danika Roy, Lisa Stoa, Shayenthiran Sreetharan, Alain Simard, Douglas R. Boreham, Joanna Y. Wilson, T.C. Tai, Simon J. Lees, Neelam Khaper

**Affiliations:** 1 Department of Science and Environmental Studies, Lakehead University, Thunder Bay, ON P7B 5E1, Canada; asnemec@lakeheadu.ca; 2 Department of Biology, Lakehead University, Thunder Bay, ON P7B 5E1, Canada; saniccol@lakeheadu.ca (S.N.); cqdavids@lakeheadu.ca (C.D.); simon.lees@nosm.ca (S.J.L.); 3 Northern Ontario School of Medicine, Laurentian University, Sudbury, ON P3E 2C6, Canada; droy5@laurentian.ca (D.R.); asimard@nosm.ca (A.S.); dboreham@nosm.ca (D.R.B.); tc.tai@nosm.ca (T.C.T.); 4 Department of Biology, McMaster University, Hamilton, ON L8S 4L8, Canada; lisastoa@mcmaster.ca (L.S.); sreeths@mcmaster.ca (S.S.); joanna.wilson@mcmaster.ca (J.Y.W.); 5 Northern Ontario School of Medicine, Lakehead University, Thunder Bay, ON P7B 5E1, Canada; 6 Biomolecular Sciences, Laurentian University, Sudbury, ON P3E 2C6, Canada

**Keywords:** low-dose radiation, cardiac, antioxidants, oxidative stress

## Abstract

Ionizing radiation (IR) is known to cause fetal programming, but the physiological effects of low-dose IR are not fully understood. This study examined the effect of low (50 mGy) to non-lethal (300 and 1000 mGy) radiation exposure during late gestation on cardiac metabolism and oxidative stress in adult offspring. Pregnant C57BL/6J mice were exposed to 50, 300, or 1000 mGy of gamma radiation or Sham irradiation on gestational day 15. Sixteen weeks after birth, ^18^F-Fluorodeoxyglucose (FDG) uptake was examined in the offspring using Positron Emission Tomography imaging. Western blot was used to determine changes in oxidative stress, antioxidants, and insulin signaling related proteins. Male and female offspring from irradiated dams had lower body weights when compared to the Sham. 1000 mGy female offspring demonstrated a significant increase in ^18^F-FDG uptake, glycogen content, and oxidative stress. 300 and 1000 mGy female mice exhibited increased superoxide dismutase activity, decreased glutathione peroxidase activity, and decreased reduced/oxidized glutathione ratio. We conclude that non-lethal radiation during late gestation can alter glucose uptake and increase oxidative stress in female offspring. These data provide evidence that low doses of IR during the third trimester are not harmful but higher, non-lethal doses can alter cardiac metabolism later in life and sex may have a role in fetal programming.

## 1. Introduction

An unfavorable fetal environment can affect growth and development of the fetus, and these changes can persist throughout life, which is commonly referred to as fetal programming. Adverse fetal environment can also result in changed placental morphology, low birth weight, and accelerated growth, which have been linked to many diseases later, including cardiovascular disease, cancer, diabetes mellitus, coronary heart disease, and hypertension [1,2,3,4,5]. Exposure to a variety of stressors during pregnancy can lead to low birth weight [6]. Stressors have the ability to activate the hypothalamic-pituitary-adrenal (HPA) axis [2,7]. Activation of the HPA axis can result in increased glucocorticoid production [2,7], which is known to cause insulin resistance and could result in altered cardiac glucose metabolism [8,9].

One type of stressor that has been shown to alter the HPA axis function is ionizing radiation (IR) [10,11]. Human exposure to high doses of radiation is uncommon outside of radiotherapy and nuclear disasters whereas low-dose exposures are more common [12]. Pregnant women can be exposed to IR from different sources, including medical diagnostic imaging, which can expose them to low levels (≤0.1 Gy) of IR. A typical CT scan can expose an individual to about 10 mSv of IR; the Canadian Nuclear Safety Commission defines 100 mSv as the lowest dose to cause cancer, and 1000 mSv is the lowest dose that may cause acute radiation syndrome in humans. 50 mSv is the annual radiation dose limit for nuclear workers in Canada [13]. According to the United Nations Scientific Committee on the Effects of Atomic Radiation (UNSCEAR), low-dose radiation is any dose below 100 mGy [12]. However, there is no consensus on what is a high versus low dose of radiation when it relates to fetal programming. Mice have been shown to be more radioresistant than humans; therefore, the dose used in rodent studies has to take into account this difference [14,15]. For the purposes of this study, 50 mGy is low whereas 300 and 1000 mGy is classified as a non-lethal dose.

While high-dose exposure to IR in mice is known to cause malformations in the developing fetus, the impact of low-dose exposure that may be comparable to doses humans receive during medical imaging is unclear. The effects of IR on fetal development depend on the time and dose received. Radiation risks are most notable in the first trimester during which organogenesis occurs, and the fetus is most susceptible to the teratogenic effects of IR, resulting in adverse effects that include growth restriction, microcephaly, and behavioral defects [16]. Radiation risks are comparatively less in the second and least in the third trimester. However, recent studies have reported that the third trimester also presents a critical gestational period whereby changes in the in-utero environment can influence the cells to change their phenotype, resulting in adaptation of cellular function that may have long-lasting implications commonly referred to as fetal programming [17]. High IR doses are known to be detrimental in mice during fetal development and during organogenesis [18,19]. This has been shown to result in growth retardation, cleft palate, altered brain development, and behavioral changes [20,21,22].

IR can be dangerous to the human body since exposure causes an increase in reactive oxygen species (ROS), and if the levels of ROS overwhelm the cells antioxidant defenses, oxidative stress can occur [23,24,25]. The absorption of IR can directly disrupt atomic structures producing chemical and biological changes or can indirectly generate ROS through the radiolysis of water [26]. Cells respond to increased ROS by upregulating the expression of cellular antioxidant defenses, and treatment with antioxidants has been shown to reduce radiation-induced oxidative damage [27,28,29]. In addition, it has been shown in animal studies that low-dose radiation (LDR) exposure activates endogenous antioxidant defense systems that can reduce the oxidative damage produced by ROS [30,31]. This adaptive response has been observed in many cellular processes, including cell survival, gene mutations, and immune response [32,33,34]. Although there is emerging evidence that LDR exposure during late gestation can cause fetal programming [35], it is unclear whether the cellular programming is mediated in part by oxidative stress, what the outcome of LDR on adult heart glucose metabolism is, and if LDR induces an adaptive antioxidant response.

Under normal conditions, the heart primarily uses free fatty acid oxidation as its major source of energy and will store glucose as glycogen to use when there is a need for high energy output [36]. During chronic stress including cardiac hypertrophy, there is a shift from fatty acid oxidation to glucose utilization, which is associated with an increase in glycolysis [37]. The increase in glycolysis may lead to an imbalance between glucose uptake and oxidation, which has been implicated in cardiac diseases associated with redox imbalance and contractile dysfunction [37]. Moreover, alterations to glucose metabolism at a young age may contribute to severe metabolic dysfunction such as diabetes and metabolic syndrome later life [38]. Insulin-induced activation of protein kinase B (Akt) provides cardioprotection by regulating a variety of downstream signaling molecules [39]. One downstream protein of interest is glycogen synthase kinase 3-β (GSK3β), which can regulate glycogen synthesis and negatively regulate cardiac hypertrophy [40,41]. It is unclear whether in utero exposure to IR can affect the adult offspring’s cardiac glucose uptake and insulin signaling proteins.

The aim of this study is to determine if there is a dose response from exposure to IR during late gestation and how IR affects postnatal adult cardiac metabolism and oxidative stress in a mouse model of fetal programming. We hypothesize that low doses of IR exposure in utero will not have adverse outcomes in the adult offspring since radiation hormesis has been shown in low doses.

## 2. Materials and Methods

### 2.1. Animal Care and Experimental Groups

Animal use protocols were approved by the Lakehead University Animal Care Committee and the Animal Research Ethics Board at McMaster University, and 7–8-week-old male and female C57BL/6J mice were obtained from Jackson Laboratory (Bar Harbor, Maine, USA). Animals were maintained on a 12:12 h light:dark cycle and allowed food and water *ad-libitum*. Housing temperature was controlled, at 20–22 °C. The mice were given one week to acclimate without disruption prior to breeding, putting the mice at 8–9 weeks of age at the time of breeding. Two female mice were moved to a cage with a single housed male (breeding was performed 2:1) and left to breed overnight. Vaginal plug was used to determine the first day of gestation, and females were singly housed after breeding. We randomized the breeding pairs in an attempt to avoid parental biases. Due to the radiation exposure, only first-time dams were used. Pregnant mice were Sham irradiated or received 50, 300 or 1000 mGy of whole-body gamma radiation (662 keV energy) using a cesium-137 source (Taylor Radiobiology Source) on gestational day 15 at McMaster University. Day 15 of gestation was chosen because previous fetal programming studies, including our own, have shown that exposure during the third trimester, usually gestational days 15 to 19, induces observable changes in offspring [42,43]. Briefly, pregnant mice were placed under the source for irradiation in their home cage for the required time and radiation was delivered at a nominal dose rate of 10 mGy/min. Sham-irradiated mice were placed under the source for 20 min (with the source off) then moved to the control room for the remainder of the longest irradiation time. All animals were restricted for food and water consumption for the duration of irradiation. The fetal dose was verified by using the thermoluminescent dosimeters as previously described by our group [44]. A maximum of 2 male and 2 female offspring from one dam was used in this study to control for any maternal biased effects. Offspring were transported to Lakehead University animal facility when they reached adulthood, and cardiac tissues and plasma were collected when the offspring reached 16–20 weeks of age and stored at −80 °C until further analysis. Offspring from each group were also injected with either saline or 0.5 U HumulinR 15 min prior to tissue collection to investigate insulin-related signaling proteins.

### 2.2. Tissue Preparation

Tissues were ground into a powder using a mortar and pestle and were kept frozen during preparation using liquid nitrogen. Frozen powder was weighed to the desired amount and was chemically lysed with Pathscan (25 mM Tris pH 7.5, 150 mM NaCl, 1mM EDTA, 1% Triton X) containing a protease inhibitor cocktail (1:100; Sigma-Aldrich, Oakville, ON, Canada), sodium orthovanadate (1:100; Abcam, Danvers, MA, USA), and sodium fluoride (40 uL/mL; Fischer Scientific, Waltham, MA, USA). The tissues were further disrupted using the Tissue Lyser (Qiagen, Redwood City, CA, USA) for 2 min at 20 Hz. Samples were then centrifuged for 10 min at 16,000× *g*, and the supernatant was stored at −80 °C. To quantify the amount of protein in each sample, the BCA protein assay (PierceTM, Waltham, MA, USA) was used. Following manufacturers protocol, the samples absorbances were compared to BSA standards to determine the concentration. Protein samples were stored at −80 °C for biochemical assays.

### 2.3. Positron Emission Tomography

Fasted glucose uptake was assessed using ^18^F-fluorodeoxyglucose (^18^F-FDG) obtained from the local cyclotron (Thunder Bay Regional Health Sciences Centre Cyclotron and Radiopharmacy, Thunder Bay, ON, Canada). Mice were moved to new cages without food 5 h before the first scan. Approximately 20 μCi of ^18^F-FDG in sterile saline was given to each mouse intraperitoneally. One hour after ^18^F-FDG injection, mice were anaesthetized with 1.5% isoflurane and were imaged in a G4 PET/X-ray scanner (Sofie Biosciences, Culver City, CA, USA). Each mouse underwent a 10 min scan, and images were analyzed using VivoQuantTM (Version 1.23, Invicro, Boston, MA, USA). Glucose uptake was reported as maximum standardized uptake value (SUVmax).

### 2.4. Glycogen Content

Cardiac glycogen content was assessed using the EnzyChromTM Glycogen assay kit (Bioassay Systems, Hayward, CA, USA) according to manufacturer’s instructions. Glycogen concentration expressed as microgram per ml was determined by using standards and by subtracting the sample blank to account for presence of glucose in the sample.

### 2.5. Antioxidant Enzyme Activity

Catalase activity was determined spectrophotometrically by using the method of Beers and Sizer as described by Aebi [45] and was expressed as nmol/min/mg of protein. This method measures the exponential breakdown of H_2_O_2_ (Sigma-Aldrich, Oakville, ON, Canada) at 240 nm in the presence of cellular homogenate. Superoxide dismutase (SOD) activity was measured spectrophotometrically as described by Marklund and was expressed as USOD/mg of protein. The autoxidation of pyrogallol (Sigma-Aldrich, Oakville, ON, Canada) is sustained by hydroxyl radical, which is inhibited by SOD. The inhibition of this process can be measured to determine SOD concentration in the sample [46]. Glutathione peroxidase (GPx) activity was measured by using the spectrophotometric method of Paglia and Valentine. 1U of GPx activity was defined as the amount of protein that oxidized 1 μmol of reduced NADPH (Sigma-Aldrich, Oakville, ON, Canada) per minute. Results are expressed as mmol/mg of protein [47].

### 2.6. NADPH Oxidase Enzyme Activity

NADPH oxidase activity was determined spectrophotometrically by the rate of NADPH consumption over 20 min at 340 nm, and the rate of the reaction was expressed as μmol/min/mg of protein (Sigma-Aldrich, Oakville, ON, Canada) [48].

### 2.7. Total ROS Content

Total amount of ROS was measured by the oxidation of the DCFDA fluorochrome (Sigma 35848), which is esterified by cellular esterase’s to DCFH, and this is oxidized to DCF in the presence of reactive species [49]. The subsequent fluorescence was measured using the FLUOrstar OPTIMA (BMG Labtech, Ortenberg, Germany) and was presented as nmol/mg of DCF.

### 2.8. Redox Ratio

A glutathione assay kit (Cayman Chemical, Ann Arbor, MI, USA) was used to measure the reduced glutathione (GSH) and oxidized glutathione (GSSG) levels as per the manufacturer’s protocol. The reaction between GSH and DTNB (5,5′-dithio-bis-2-nitrobenzoic acid) results in a coloured product TNB (5-thio-2-nitrobenzoic acid). The absorbance of TNB was measured at 405 nm spectrophotometrically. Glutathione redox ratio was expressed as a ratio of GSH to GSSG.

### 2.9. Western Blot

Protein from cardiac tissue samples were boiled at 95 °C for 5 min in Laemmli buffer and subjected to SDS-page electrophoresis. Proteins were transferred to a nitrocellulose membrane using a Trans-blot apparatus (Bio-Rad, Mississauga, ON, Canada). The membrane was blocked with 5% milk for 1 h. Membranes were then incubated with the appropriate primary antibody overnight at 4 °C. Following washing in 1× Tween-Tris buffered saline (TBST; pH 7.4), the membrane was incubated with the appropriate horseradish peroxidase conjugated secondary antibody for 1 h. Following chemiluminescent imaging, the membrane was stripped and re-blotted accordingly. ImageJ software was used to quantify band density. Ponceau S (Fisher Scientific, Branchburg, NJ, USA) staining was used to demonstrate equal loading. Several heart lysates were repeated across gels as loading controls. The dilutions of the antibodies were as follows: pAkt/panAkt (1:1000, Cell Signaling Technologies, Danvers, MA, USA), pGSK3β/tGSK3β (1:1000, Cell Signaling Technologies, Danvers, MA, USA), anti-catalase (1:1000; Sigma-Aldrich, Oakville, ON, Canada), anti-MnSOD (1:2500; Millipore, Billerica, MA, USA), and anti-GPx (1:5000; Abcam, Danvers, MA, USA). Anti-rabbit IgG (1:1000–1:5000; Millipore, Burlington, MA, USA) secondary antibody was used for all primary antibodies except for catalase, for which we used anti-mouse IgG (1:4000; Millipore, Burlington, MA, USA).

### 2.10. Plasma Cytokine Concentration

Plasma cytokine (interleukin (IL)-1β, IL-10, IL-6, tumour necrosis factor alpha (TNF-α), and IL-12p70) levels were determined by using the BD cytometric bead array (CBA) Flex Set kits (BD Pharmingen, San Diego, CA, USA). Briefly, samples were prepared by following the manufacturers protocol, then subjected to flow cytometry (BD FACS Canto II), and samples were analyzed using FCAP Array v3.

### 2.11. Statistical Analysis

Data was presented as the mean ± standard error of the mean (SEM). Data was analyzed using the one way-ANOVA test and the Fisher’s LSD post hoc test (GraphPad Prism 6 software, San Diego, CA, USA). A two way-ANOVA was used to analyze experiments using insulin as a second variable. Comparisons with *p*-values ≤ 0.05 were considered significantly different.

## 3. Results

### 3.1. Body and Heart Weight

Offspring were weighed before being euthanized at 16–20 weeks old. Male body weights from the 50 mGy and 1000 mGy groups were significantly decreased as compared to the Sham and 300 mGy groups (Table 1). Male heart weights were significantly lower in the 1000 mGy group compared to the Sham and 300 mGy groups, but when normalized to body weight there were no differences in heart weight between groups (Table 1). Female offspring in the 50, 300, and 1000 mGy groups had significantly reduced body weights compared to Sham group. 50 mGy female offspring heart weights were significantly lower than Sham, and 1000 mGy was significantly lower than Sham and 300 mGy groups (Table 1).

### 3.2. Cardiac Glucose Uptake, Storage, and Insulin Signaling

**Figure 1:**
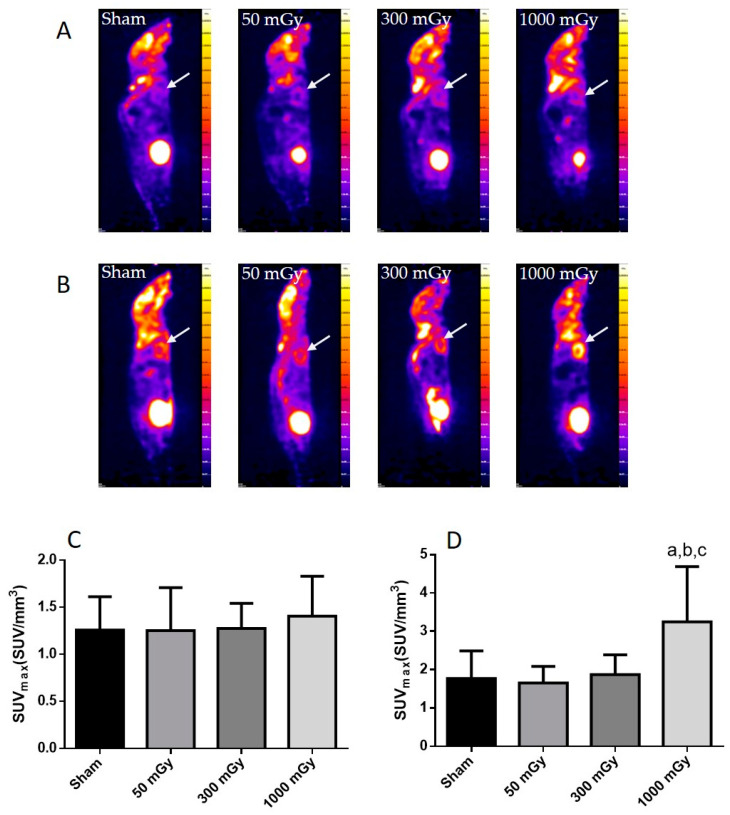
Cardiac glucose uptake in mice after receiving Sham, 50, 300, or 1000 mGy gamma radiation in utero. Representative PET scans demonstrating ^18^F-FDG uptake in male (**A**) and female (**B**) offspring cardiac tissue as denoted by the white arrow. Quantitative analysis for ^18^F-FDG PET image measured as maximum standardized uptake value (SUVmax) in males (**C**) and females (**D**). Data are expressed as mean ± SEM. a—significantly different to Sham, b—significantly different to 50 mGy, c—significantly different to 300 mGy, and *p* ≤ 0.05. *n* = 11–19 per group.

**Figure 2:**
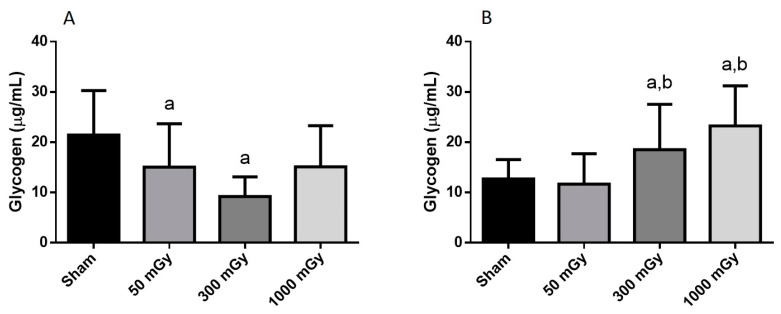
Cardiac glycogen content. (**A**) male and (**B**) female offspring cardiac glycogen levels. Data are expressed as mean ± SEM. a—is significantly different as compared to Sham, and b—is significantly different as compared to 50 mGy, *p* ≤ 0.05. *n* = 11–19 per group.

**Figure 3:**
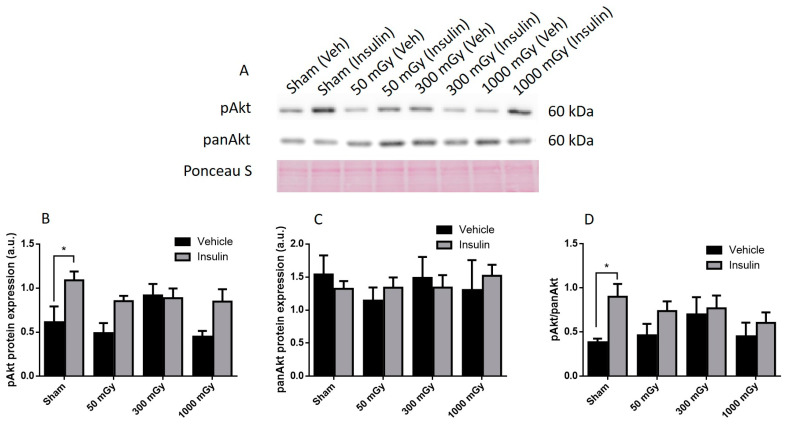
Representative western blot (**A**) and quantified band density (relative protein expression in arbitrary units) for phosphorylated Akt (pAkt) (**B**), Pan Akt (panAkt) (**C**), and the ratio of phosphorylated protein to total protein (**D**) in hearts of male offspring. Results were normalized to loading controls (samples repeated on each gel) to account for differences between gels and Ponceau S staining was used as a marker of equal protein loading. Data are presented as mean ± SEM. *, denotes significantly different between vehicle and insulin injection, and *p* ≤ 0.05. *n* = 11–13 per group.

**Figure 4:**
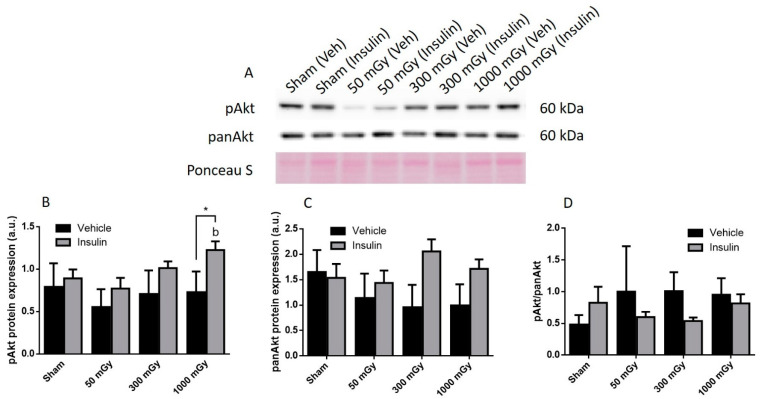
Representative western blot (**A**) and quantified band density (relative protein expression in arbitrary units) for phosphorylated Akt (pAkt) (**B**), Pan Akt (panAkt) (**C**), and the ratio of phosphorylated protein to total protein (**D**) in female offspring hearts. Results were normalized to loading controls (samples repeated on each gel) to account for differences between gels, and Ponceau S staining was used as a marker of equal protein loading. Data are presented as a mean ± SEM. *, denotes significantly different between vehicle and insulin injections, b—is significantly different from 50 mGy group, and *p* ≤ 0.05. *n* = 11–19 per group.

**Figure 5:**
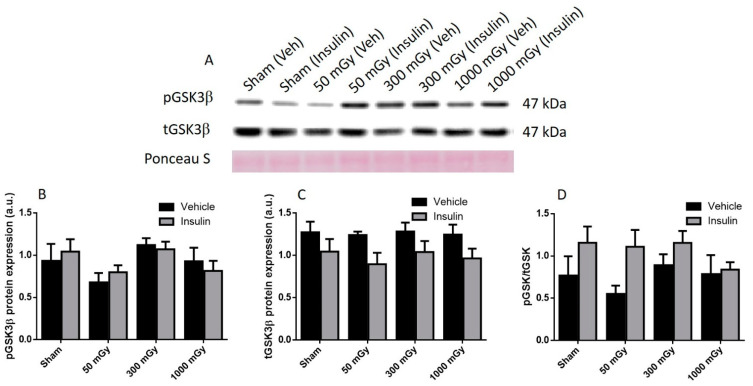
Representative western blot (**A**) and quantified band density (relative protein expression in arbitrary units) for phosphorylated GSK3β (pGSK3β) (**B**), total GSK3β (tGSK3β) (**C**), and the ratio of phosphorylated protein to total protein (**D**) in male offspring hearts. Results were normalized to loading controls (samples repeated on each gel) to account for differences between gels, and Ponceau S staining was used as a marker of equal protein loading. Data are presented as a mean ± SEM. *n* = 11–13 per group.

**Figure 6:**
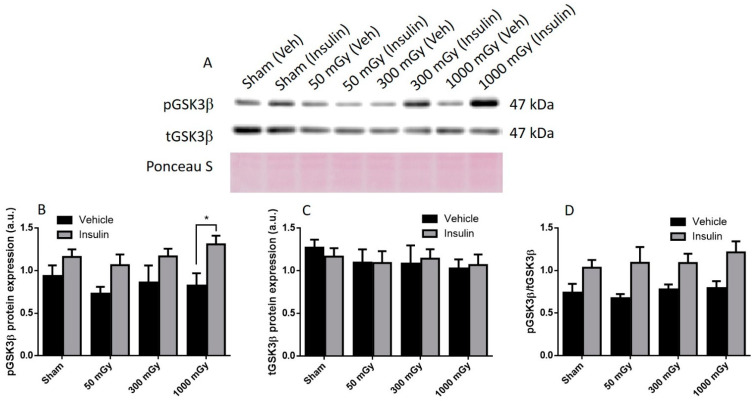
Representative western blot (**A**) and quantified band density (relative protein expression in arbitrary units) for phosphorylated GSK3β (pGSK3β) (**B**), total GSK3β (tGSK3β) (**C**), and the ratio of phosphorylated protein to total protein (**D**) in hearts of female offspring. Results were normalized to loading controls (samples repeated on each gel) to account for differences between gels, and Ponceau S staining was used as a marker of equal protein loading. Data are presented as a mean ± SEM. *, denotes significantly different between vehicle and insulin injections, and *p* ≤ 0.05. *n* = 11–19 per group.

Offspring underwent PET imaging at 16–20 weeks of age to measure glucose uptake. There was no significant difference in cardiac glucose uptake between male offspring exposed to different doses of IR in utero (Figure 1A–C). In the female offspring, the 1000 mGy group had significantly higher cardiac glucose uptake than all three other groups (Figure 1B,D). The heart primarily converts glucose to glycogen and stores it for when the heart needs a burst of energy [37]. We therefore determined if glucose uptake resulted in increased glycogen stores. Glucose storage as glycogen in male offspring was significantly lowered in 50 mGy and 300 mGy IR groups compared to Sham (Figure 2A). Glycogen was significantly increased in the 300 mGy and 1000 mGy groups compared to both Sham and 50 mGy female offspring (Figure 2B). Following insulin stimulation, phosphorylation and expression of Akt and GSK3β signaling proteins were measured to determine if insulin signaling was affected by IR. The presence of insulin can influence the amount of glucose being taken up in the heart [50,51]. Between radiation groups, phosphorylated Akt at SER473 (pAkt), panAkt (total Akt), and the ratio of pAkt/panAkt were not changed in the male heart tissues (Figure 3A–C). Insulin increased pAkt compared to the vehicle injection only in the Sham group (Figure 3A). In female offspring heart tissues, pAkt was only significantly increased in the 1000 mGy group compared to 50 mGy when insulin stimulated (Figure 4A) as well as compared to its respective vehicle group. panAkt and the ratio of pAkt/panAkt were not altered from fetal IR exposure (Figure 4B,C). Phosphorylated and total GSK3β protein expression was not significantly changed in either male or female heart tissues (Figure 5 and Figure 6). Insulin only increased pGSK3β in the 1000 mGy group compared to vehicle injection (Figure 6A).

### 3.3. Antioxidant and Oxidative Stress Status

**Figure 7:**
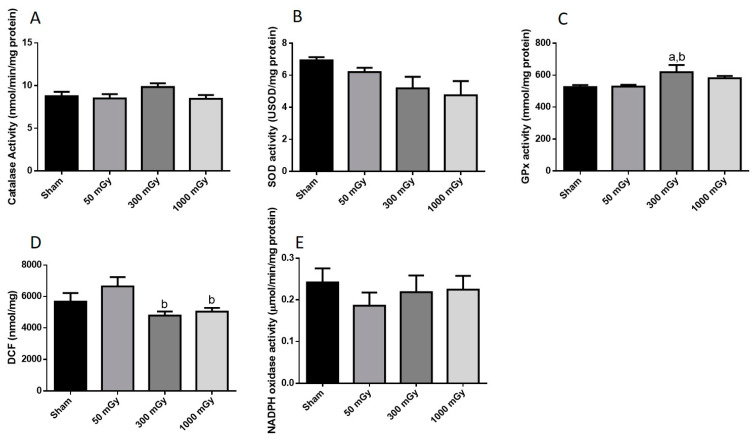
Antioxidant and oxidative stress status. Enzyme activity of the antioxidants (**A**) catalase, (**B**) superoxide dismutase (SOD), and (**C**) glutathione peroxidase (GPx); and (**D**) total reactive oxygen species expressed as mmol/mg of DCF; and (**E**) NADPH oxidase activity in male offspring heart tissues. Data are presented as a mean ± SEM. a—is significantly different than control, and b—is significantly different than 50 mGy, *p* ≤ 0.05. *n* = 11–13 except (**E**), *n* = 7–13 per group.

**Figure 8:**
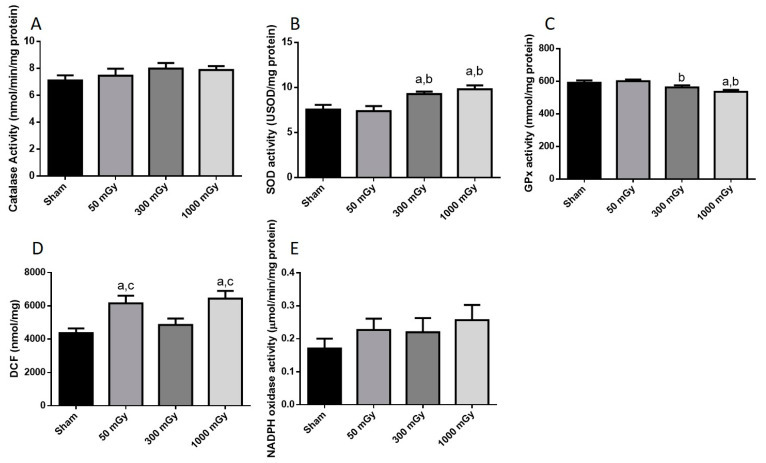
Antioxidant and oxidative stress status. Enzyme activity of the antioxidants (**A**) catalase, (**B**) superoxide dismutase (SOD), and (**C**) glutathione peroxidase (GPx); and (**D**) total reactive oxygen species expressed as mmol/mg of DCF; and (**E**) NADPH oxidase activity in female offspring heart tissues. Data are presented as a mean ± SEM. a—is significantly different than control, b—is significantly different than 50 mGy, and c—is significantly different from 300 mGy, *p* ≤ 0.05. *n* = 11–19 except (**E**) where *n* = 8–9 per group.

**Figure 9:**
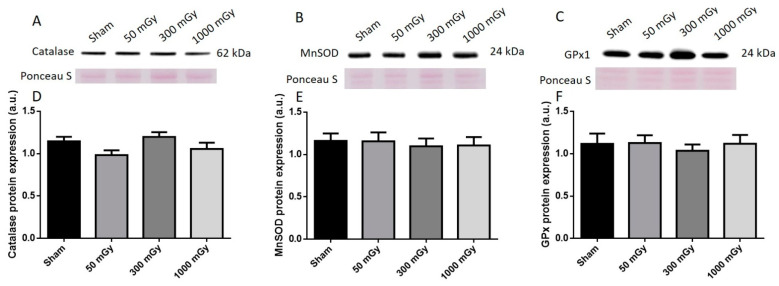
Representative western blot (**A**–**C**) and quantified band density (**D**–**F**) (relative protein expression in arbitrary units) for (**A**) catalase, (**B**) manganese superoxide dismutase (MnSOD), and (**C**) glutathione peroxidase 1 (GPx1) in male offspring cardiac tissues. Results were normalized to loading controls (samples repeated on each gel) to account for differences between gels, and Ponceau S staining was used as a marker of equal protein loading. Data are presented as a mean ± SEM. *n* = 11–13 per group.

**Figure 10:**
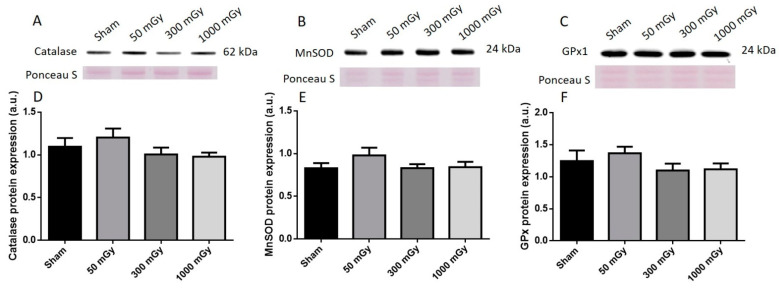
Representative western blot (**A**–**C**) and quantified band density (**D**–**F**) (relative protein expression in arbitrary units) for (**A**) catalase, (**B**) manganese superoxide dismutase (MnSOD), and (**C**) glutathione peroxidase 1 (GPx1) in female offspring cardiac tissues. Results were normalized to loading controls (samples repeated on each gel) to account for differences between gels, and Ponceau S staining was used as a marker of equal protein loading. Data are presented as a mean ± SEM. *n* = 11–19 per group.

IR has been shown to increase the production of ROS, which can lead to the damage of proteins and DNA [26]. Oxidative stress is also associated with metabolic dysfunction, and studies have shown that oxidants can result in impaired insulin mediated glucose uptake [52]. Endogenous antioxidant activity was measured to determine if prenatal exposure to IR during the last trimester would induce an adaptive antioxidant response to the expected increase in ROS. Catalase and SOD enzyme activities were not altered due to prenatal IR exposure in male offspring (Figure 7A,B). GPx activity was increased in the 300 mGy group compared to Sham and 50 mGy groups (Figure 7C). Total ROS level was decreased in the 300 mGy and 1000 mGy groups compared to the 50 mGy group in male offspring (Figure 7D). NADPH oxidase (one of the major sources of ROS) activity was not changed due to IR exposure in any of the groups (Figure 7E). In female offspring, catalase activity was not altered by IR exposure (Figure 8A). SOD activity was significantly increased in the 300 mGy and 1000 mGy groups compared to the Sham and 50 mGy groups (Figure 8B). GPx activity was significantly reduced in the 300 mGy group compared to the 50 mGy group, and the 1000 mGy group was significantly reduced compared to the Sham and 50 mGy groups (Figure 8C). Total ROS level was increased in 50 mGy and 1000 mGy groups compared to the Sham and 300 mGy groups (Figure 8D). NADPH oxidase activity was not significantly increased after IR exposure but did seem to have a trend to increase as IR dose increased (Figure 8E). Redox ratio measured as GSH/GSSG was used as a marker of oxidative stress where an increase in the ratio indicates increased oxidative stress and vice versa. In male offspring, there was no significant change in reduced or oxidized glutathione levels or the redox ratio (Table 2). In female offspring, GSSG was significantly increased in 300 mGy group compared to Sham (Table 2). GSSG was also significantly increased in the 1000 mGy group compared to all other groups (Table 2). GSH/GSSG was significantly decreased in the 300 mGy group compared to Sham and 50 mGy groups, and the 1000 mGy group was significantly different from all other groups (Table 2). Altered antioxidant enzyme activity was not accompanied by a change in the antioxidant protein expression in either male or female heart tissues (Figure 9 and Figure 10).

### 3.4. Circulating Cytokines

**Figure 11:**
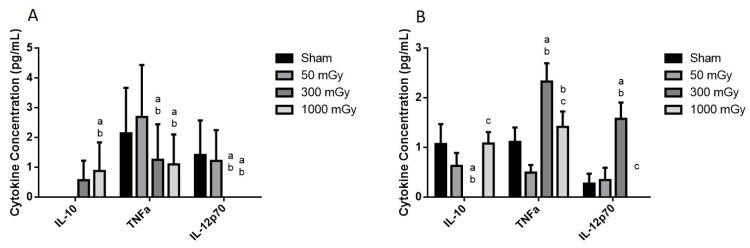
Plasma cytokine concentration in offspring after being exposed to IR in utero. (**A**) male and (**B**) female plasma concentrations of cytokines. Data are presented as a mean ± SEM. a—is significantly different from Sham, b—is significantly different from 50 mGy, and c—is significantly different from 300 mGy, *p* ≤ 0.05. *n* = 11–19 per group.

Plasma cytokines were assessed to determine if prenatal exposure to IR was able to induce an immune response in adult offspring. IL-10 was significantly increased in the 1000 mGy male group when compared to Sham and 50 mGy groups (Figure 11A). TNFα and IL-12p70 were significantly lower in the male 300 mGy and 1000 mGy groups when compared to Sham and 50 mGy (Figure 11A). In both male and female offspring, IL-1β and IL-6 were undetectable. TNFα was significantly increased in the 300 mGy group compared to all other groups and in the 1000 mGy compared to 50 mGy group (Figure 11B). IL-12p70 was significantly increased in the 300 mGy group compared to Sham and 50 mGy groups while was undetectable in the 1000 mGy group (Figure 11B).

## 4. Discussion and Conclusions

An adverse fetal environment can result in programming that causes developmental changes that can persist through to adulthood. IR exposure during fetal development is known to cause malformations and growth restriction when exposed during organogenesis [53]. IR exposure in human fetuses during late gestation has been shown to cause growth restriction, reduced brain function, and miscarriage at high doses, but exposure below 50 mGy shows no adverse health effects to the fetus [17,53,54,55]. Moreover IR produces oxidative stress, suggesting that there is a potential role for IR in developmental programming due to an overlapping mechanism of action [16].

In our study, cardiac glucose uptake, glycogen storage, insulin signaling proteins, and oxidative stress markers were assessed in 16–20 weeks-old male and female offspring after receiving low to non-lethal doses of IR in utero during late gestation. The results of our study demonstrate that non-lethal radiation (300–1000 mGy) affected glucose uptake, storage, and antioxidant potential in female offspring but did not have the same effect at low doses (50 mGy) or in male offspring. The significant differences between males and females seen in this study are not entirely unexpected based on previous literature that reported sex differences in the fetal programming of cardiovascular and metabolic function in both animal and human studies [56,57,58]. Although the underlying mechanism of the sex differences remains unknown, it can be speculated that differences in sex hormone concentrations between males and females may play a role. In this regard, a modulatory role of testosterone on glucose metabolism has also been reported [59].

Maternal stress during pregnancy has been shown to cause low birth weight, which is associated with cardiovascular disease and type-2 diabetes [60]. Radiation therapy has also been associated with cardiovascular disease as a long term side effect [61,62]. The long-term effects of fetal LDR exposure on the cardiovascular system have yet to be fully understood. In this study, the 1000 mGy dose IR exposure resulted in lower overall adult body weight. In males, the 1000 mGy group had reduced heart weight, but this was accompanied by lower body weight. We have previously reported a reduction in body weight in the 1000 mGy group [44]. Previous studies have reported a correlation between low birth weight or restricted growth in offspring and an increased incidence of cardiovascular disease in adulthood. A low birth weight at higher doses is a well-documented phenomenon of fetal programming. We did not measure birth weights of the offsprings and maternal body weights prior to weaning to reduce handling stress and prevent cannibalism. Previously, decreased heart weight has been observed in a rat model of fetal programming where there were no differences in the heart weight to body weight ratio [63].

Fasted glucose uptake was not altered by IR in male offspring but was increased in female offspring 1000 mGy group. We have also previously reported increased ^18^F-FDG uptake in brown adipose tissue in female offspring of irradiated dams [42]. 1000 mGy female offspring also demonstrated an increase in glycogen stores, whereas males showed a significant decrease in the 50 and 300 mGy groups. Both glucose uptake and glycogen content remained unchanged in the 1000 mGy male offspring compared to the Sham group. Future Investigation into the role of regulatory enzymes involved in gluconeogenesis may provide some insight regarding these differences. Altered glucose uptake and glycogen stores have been associated with cardiac hypertrophy resulting in impaired cardiac function [37]. Depletion of glycogen stores has also been associated with impaired contractility and relaxation [36]. Previous studies have shown sex differences in glucose uptake and utilization where women were more insulin-sensitive [64] and had reduced glucose utilization than males [65]. Foryst-Ludwig et al., (2011) demonstrated reduced glucose uptake in female mice, while glucose uptake did not change in male mice after exercise [66]. Moreover, the difference in cardiac glucose uptake between the two sexes was not accompanied by a change in glycogen content. Furthermore, some studies have reported that females are more radiosensitive than males [67,68], which may explain the difference in glucose metabolism between male and female offsprings in our study. Pogribny et al. have also demonstrated that the effects of radiations are sex-, dose-, and tissue-dependent [69]. Insulin regulates glucose uptake by binding to the insulin receptors, eventually resulting in the phosphorylation of Akt [48,70]. In this study, data from male offspring showed that insulin had an effect on Sham mice, but the mechanism behind unchanged levels in radiation group remains to be investigated in the future. This study also demonstrated that the increase in glucose uptake and glycogen stores is not related to the Akt signaling pathway in female offspring.

IR causes the formation of ROS, and in excess can cause DNA, cell membrane, and organelle damage [26]. Oxidative stress is also associated with metabolic dysfunction, and studies have shown that oxidants can result in impaired insulin mediated glucose uptake [52,71]. Low to non-lethal radiation exposure during late gestation demonstrated an increase in the GPx activity in the 300 mGy group compared to Sham and 50 mGy groups as well as a decrease in total ROS level in the 300 mGy and 1000 mGy groups in male offspring cardiac tissues. However, female offspring cardiac tissues demonstrated evidence of redox imbalance. Increase in the SOD activity and decrease in the redox ratio suggest that the antioxidant defense system is upregulated in female 1000 mGy group. The altered redox ratio could be related to the decrease in GPx activity. While SOD is the first line of defense against ROS mediated damage, GPx is an important antioxidant enzyme in the heart and in the presence of GSH, GPx catalyzes the reduction of peroxides [72]. In the 1000 mGy group, the concentration of GSSG is nearly doubled compared to Sham, suggesting increased ROS scavenging by glutathione in that group compared to the Sham mice. This suggests that cardiac redox homeostasis is sex-specific and may depend on the degree of oxidative stress. Some studies also suggest that there may be delayed effects from IR in the heart [73]. In this regard, the antioxidant response demonstrated here may eventually be exhausted, and oxidative stress could still occur resulting in disease much later in life [74]. Increased ROS and the depletion of endogenous antioxidant defense system have been reported in heart diseases, including hypertension and diabetes [74,75]. Taken together, the changes in antioxidants status and glucose metabolism at 16–20 weeks of age may indicate the beginning of cardiac dysfunction [37,76].

Low to non-lethal radiation exposure during late gestation did not elicit a robust immune response, although some of the cytokines were affected in the 300 and 1000 mGy groups. All cytokines were just above or below detection limits of the commercially available kits used. Cytokine concentrations in healthy mice are normally very low or undetectable by ELISA kits that are currently available, unless the immune system has been stimulated [77,78]. Recent studies focused on studying low-dose radiation effects found there was no change in lymphocyte populations and only slight changes in secreted cytokines as well [79,80]. Therefore, our model of IR-induced fetal programming did not increase cytokines present in adult offspring plasma to a level that would induce adverse effects. In addition to assessing plasma cytokines, it would be valuable to determine if there was immune cell infiltration in the heart tissue in future studies to determine the exact source of the cytokines.

To our knowledge, this is the first-time cardiac glucose metabolism and oxidative stress have been investigated in the offspring of dams exposed to low to non-lethal IR during late gestation, and we have demonstrated that there are sex differences. This study further adds to the evidence that low-dose radiation (50 mGy) does not have any harmful effects on the offspring when receiving radiation in utero during late gestation, and that 1000 mGy may be a threshold dose for sex-specific alterations in cardiac glucose metabolism and oxidative stress in fetal programming. Therefore, better characterization of the effects of prenatal radiation exposure will aid in our understanding of the risks associated with radiation exposure during pregnancy.

## Figures and Tables

**Table 1:** Male and female body, heart weights, and heart/body ratios in offspring after receiving Sham, 50, 300, or 1000 mGy gamma radiation in utero.

	Sham	50 mGy	300 mGy	1000 mGy
Male Body Weight (g)	30.63 ± 0.83	28.11 ± 0.45 ^a^	30.47 ± 0.82 ^b^	26.79 ± 0.57 ^ac^
Female Body Weight (g)	22.95 ± 0.63	21.26 ± 0.93 ^a^	21.69 ± 0.34 ^a^	20.71 ± 0.53 ^a^
Male Heart Weight (mg)	118.3 ± 3.46	115.7 ± 2.65	123.7 ± 2.95	109.3 ± 3.19 ^ac^
Female Heart Weight (mg)	98.82 ± 2.43	92.17 ± 1.47 ^a^	96.23 ± 1.62	89.79 ± 1.63 ^ac^
Male Heart/Body Weight (mg/g)	3.87 ± 0.071	4.11 ± 0.11	4.08 ± 0.084	4.08 ± 0.063
Female Heart/Body Weight (mg/g)	4.31 ± 0.067	4.34 ± 0.056	4.44 ± 0.063	4.35 ± 0.062

Data are expressed as mean ± SEM. ^a^—is significantly different than Sham, ^b^—is significantly different than 50 mGy, ^c^—is significantly different than 300 mGy, and *p* ≤ 0.05. *n* = 11–19 per group.

**Table 2:** Cardiac reduced (GSH) and oxidized glutathione (GSSG) levels, and redox ratio (GSH/GSSG) in male and female offspring after being exposed to 50 mGy, 300 mGy, or 1000 mGy gamma radiation in utero.

	Sham	50 mGy	300 mGy	1000 mGy
Male GSH (μM)	106.2 ± 4.15	114.3 ± 18.72	100.2 ± 9.62	105.3 ± 6.94
Male GSSG (μM)	60.97 ± 4.82	66.75 ± 7.42	55.88 ± 4.88	60.72 ± 3.76
Male GSH/GSSG	1.84 ± 0.15	1.96 ± 0.33	1.955 ± 0.26	1.80 ± 0.15
Female GSH (μM)	96.76 ± 5.57	98.32 ± 5.32	83.4 ± 4.86	86.68 ± 6.94
Female GSSG (μM)	35.13 ± 3.034	42.24 ± 3.79	55.57 ± 5.67 ^a^	86.82 ± 5.62 ^abc^
Female GSH/GSSG	2.95 ± 0.30	2.56 ± 0.37	1.779 ± 0.21 ^ab^	1.05 ± 0.10 ^abc^

^a^—is significantly different from Sham, ^b^—is significantly different from 50 mGy, and ^c^—is significantly different from 300 mGy, *p* ≤ 0.05. *n* = 11–19 per group. Data are presented as a mean ± SEM.

## Data Availability

All relevant data are within the manuscript files.
